# Evolutionary Innovations and the Organization of Protein Functions in Genotype Space

**DOI:** 10.1371/journal.pone.0014172

**Published:** 2010-11-30

**Authors:** Evandro Ferrada, Andreas Wagner

**Affiliations:** 1 Department of Biochemistry, University of Zurich, Zurich, Switzerland; 2 The Santa Fe Institute, Santa Fe, New Mexico, United States of America; 3 Swiss Institute of Bioinformatics, Lausanne, Switzerland; 4 Department of Biology, University of New Mexico, Albuquerque, New Mexico, United States of America; Centre for Genomic Regulation (CRG), Universitat Pompeu Fabra, Spain

## Abstract

The organization of protein structures in protein genotype space is well studied. The same does not hold for protein functions, whose organization is important to understand how novel protein functions can arise through blind evolutionary searches of sequence space. In systems other than proteins, two organizational features of genotype space facilitate phenotypic innovation. The first is that genotypes with the same phenotype form vast and connected genotype networks. The second is that different neighborhoods in this space contain different novel phenotypes. We here characterize the organization of enzymatic functions in protein genotype space, using a data set of more than 30,000 proteins with known structure and function. We show that different neighborhoods of genotype space contain proteins with very different functions. This property both facilitates evolutionary innovation through exploration of a genotype network, and it constrains the evolution of novel phenotypes. The phenotypic diversity of different neighborhoods is caused by the fact that some functions can be carried out by multiple structures. We show that the space of protein functions is not homogeneous, and different genotype neighborhoods tend to contain a different spectrum of functions, whose diversity increases with increasing distance of these neighborhoods in sequence space. Whether a protein with a given function can evolve specific new functions is thus determined by the protein's location in sequence space.

## Introduction

During more than half a century of protein research, an enormous amount of data about protein sequences, their structures, and their functions has accumulated. To organize the vast number of known protein sequences, the concept of a sequence space is useful [1]. Two sequences in this space have a distance, which can be measured in various ways [2], [3]. The simplest such measure is the sequence distance, the number or percentage of amino acid changes needed to transform one protein onto another. Two sequences in this space can have either the same or a different fold. This fold is the three-dimensional arrangement of their amino acids, and typically involves a specific arrangement of α-helices and/or β-sheets, the secondary structure elements of proteins. The organization of protein structures in sequence space has several general features.

First, only a small fraction of protein sequences, perhaps no larger than 10^−4^, may adopt a stable, well-defined structure [4]. Considering the astronomical size of sequence space, however, this still leaves many proteins that fold. For example, for proteins of length 100 amino acids, sequence space has 20^100^ members. Even if only one in 10^4^ of them adopts a stable structure, approximately 10^126^ foldable sequences exist in this space.

Second, the existing repertoire of protein folds is small [5], [6], and the number of sequences greatly surpasses its size.

Third, many of a protein's immediate neighbors – sequences differing from it in a single amino acid – typically have the same fold as the protein itself [7]–[9].

Fourth, even very distant sequences can have the same fold [10], [11]. If two such sequences have the same common ancestor, they are often referred to as members of the same *protein family* [6]. Such unambiguous common ancestry can usually be identified for sequences that differ in up to 60 to 70 percent of their amino acids [12]. Two sequences in the same family can be connected through a series of amino acid changes that traverse a fraction of sequence space while leaving the structure unchanged. When common ancestry can be claimed based on criteria such as common aspects of structure or function, families of proteins are grouped into superfamilies. Superfamilies share a common fold and diverge on average around 70 to 80 percent in sequence space. Sets of superfamilies that share the same three-dimensional arrangement of secondary structure are grouped into the same fold. Amino acid sequences with the same fold can be very different. Based on a systematic comparison of many divergent sequences with shared folds, Rost [11] observed that such sequences can have more than 95 percent divergence.

Fifth, the number of sequences per fold may vary widely. For example, mutagenesis experiments suggest that the amino acid sequences forming an enzyme with the same structure and function as chorismate mutase may occupy a fraction 10^−23^ of sequence space [13], whereas sequences forming a functional β-lactamase domain occupy merely one 10^−64th^ of sequence space [14]. Structures adopted by many sequences are commonly called highly designable [15], [16]. There has been increasing interest in highly designable proteins due to their use as ‘scaffolds’ in the design of new protein functions [17]. One remarkable example is the zinc finger domain, which is robust to point mutations in alanine scanning experiments [18], and has proven useful in designing new DNA binding proteins [19].

Taken together, these observations suggest that the protein sequences adopting the same structure form connected networks of sequences that can reach far through sequences space and that have varying size. These properties are not only observed for real proteins, but also for lattice proteins, and other generic models of protein folding [15], [20]–[23]. They emerge from generic physicochemical properties of the protein folding process. In other words, they are characteristic of the mapping between genotypes (sequences) and phenotypes (structures) that exists for proteins. We will call a connected network of sequences with the same structure a *genotype network*.

Similar to information about protein structures, which is abundant, thousands of proteins have known and well-characterized *functions*. However, while several authors studied the distribution of structures in sequence space [22], [24]–[25], we know much less about how functions are distributed through sequence space. This question is the main focus of our work.

The need to assign a function to newly identified protein sequences has driven research into the conservation of protein functions as sequences diverge. Several studies using methods of sequence comparison agree that functional conservation is common if two proteins possess more than 50% sequence identity [26]–[30]. For gene ontology functional annotations, more than 90 percent of protein pairs over 50% sequence identity have the same function [31]. However, a study dissenting from the conclusion of earlier work found that fewer than 30 percent of proteins with more than 50 percent sequence identity have identical enzymatic functions [32].

Information like this makes it clear that we cannot simply extrapolate from structure to function. To be sure, some proteins, such as oxygen-binding globins have the same structure and function, despite great sequence divergence [10]. However, other proteins have the same structure but different functions. Examples include proteins with the TIM-barrel fold, which is associated with many enzymatic functions [33]. In addition, many functions can be carried out by proteins with different structures. Examples include DNA polymerases, which use similar catalytic mechanisms, but diverse structures, to replicate DNA [34].

Taken together, these observations show that the relationship between sequence, structure, and function is complex. Thus, any analysis aiming to understand the organization of protein functions in sequence space must not tie itself too closely to protein structure, while respecting that structure constrains function. The biggest obstacle to such an analysis is to describe and categorize protein functions for many proteins. We circumvent this obstacle by focusing on enzymes, proteins for which a well-established, albeit imperfect, functional classification exists.

To understand how protein functions are organized in sequence space is important for at least three reasons. First, it may help guide the development of methods for protein function annotation (which is not our focus here). Second, it may help identify functions that can be performed by a large number of sequences. Experimental evidence suggests that different functions may differ by orders of magnitude in the numbers of proteins that perform them [13], [14], [35], hinting that protein functions may differ in their designability just like structures do. Being able to distinguish functions that are adopted by many proteins from those adopted by few proteins would help identify functions that are easily created or modified through directed evolution experiments and rational protein engineering. Third, and most important, it may shed light on one of the key unsolved problems in evolutionary biology, namely how new functions arise in evolution. Proteins are ideal systems for systematic studies of biological systems' ability to innovate. The reason is that we already have so much information about them.

In a variety of biological systems, the existence of extended genotype networks facilitates the evolution of novel phenotypes [36]–[38]. The reason is that different regions of genotype space contain different kinds of new phenotypes. Such phenotypes can be encountered through (neutral) exploration of a genotype network and its neighborhood in sequence space. We do not know whether the same holds for proteins, that is, whether different regions of protein genotype space contain proteins with different novel functions.

To address the issues we just discussed, we use a large dataset of protein sequences with known function and structure. Our analysis uses the concept of a protein's neighborhood in sequence space, a region comprising all sequences up to some maximal distance from the protein. We show that different neighborhoods in protein sequence space contain different functions. We discuss the implications of this observation, the limitations of our procedure, and propose a general perspective on the organization of protein functions in sequence space.

## Methods

### Protein sequences. Structural and functional annotation

We obtained protein sequences from Uniprot [39]. Specifically, we used the dataset compiled in UniProtKB/Swiss-Prot that corresponds to manually curated protein sequences. By September 2009, this dataset was composed of 495,880 sequences for which experimental details and computed features were available. To facilitate protein comparison, we restricted our study to single domain proteins longer than 50 amino acids. The structural information we used is based on the CATH classification of protein structure domains (v.3.2.0) [40]. Throughout, we use the concepts of structure and domain interchangeably and define it at the level of homologous superfamily.

We mapped domains to Uniprot sequences using HMM libraries from CATH and the software HMMER [41], assigning domains to sequences at an e-value of 0.001. Using this procedure, we found a total of 174,853 single domain sequences. Because we aimed at a broad characterization of sequence space, we did not filter our dataset for redundant sequences, but simply restricted the allowed sequence identity between pairs of sequences to at most 99 percent, thus obtaining a dataset of 136,677 sequences. We discarded sequences tagged with any of the keywords: “putative”, “probable”, “by homology”. As a source of functional annotation, we used the Enzyme Nomenclature Database (EC) [42]. Since the EC classification distinguishes four different hierarchical levels of enzyme function, we used only EC assignations that possess numerical descriptors for all of the 4 levels of the hierarchy. Using information in this database, we arrived at our final data set, which comprises 39,529 protein sequences. These sequences correspond to 1,343 enzyme types classified under the EC system. They adopt 457 different structures, as indicated by their CATH domains.

Our next goal was to align sequences in our data set, in order to estimate their pairwise distance in sequence space. To do so, we grouped our sequences according to the CATH domains they had. For each sequence, we kept only the regions for which HMM profiles had detected significant sequence similarity between sequences. This procedure discards uninformative regions of proteins and improves the quality of the subsequent alignments, which we carried out with ClustalW [43]. We also tested the performance of structural alignments using T-coffee [44] and found that in the case of our dataset, Clustalw and T-coffee produced similar results. The number of sequences per multiple sequence alignment varied according to domains, with a median of 12 sequences per alignment. For further analyses we included only proteins where, after multiple sequence alignment, at most 10 percent of positions were gaps, and no more than 10 percent of any one amino acids sequence contained gaps.

We carried out two different analyses of our data. First, we characterized, for proteins with a given structure, how their functions were distributed across sequence space. To this end, we focused on 36 different structures for which at least 10 sequences are known. Specifically, these structures have between 10 and 4,132 associated sequences. Except for the TIM barrel, we carried these analyses out exhaustively, that is, considering all possible pairwise comparisons between sequences that share a structure domain (see figure legends for details). Second, we examined the distribution of functions regardless of the structures performing them. In this analysis, a complication is that proteins with different structures can have different lengths. To facilitate their embedding in the same genotype space, we focused only on alignments with sequences no shorter than 100 amino acids. The resulting (reduced) data set had 28,862 sequences, 337 different structures, and 1,036 enzyme functions. We then selected random sections of 100 residues from each multiple sequence alignment, calculated the desired statistic from the resulting resampled data, and repeated this resampling and calculation procedure a total of 10 times. (Since proteins with more than 10 percent of gaps are discarded, each one of the 10 samples comprises on average 28,862 sequences, 337 different structures, and 1,036 enzyme functions.) We performed the neighborhood analysis described below on each of these 10 samples, and report results as means and standard deviations over these 10 samples.

## Results

To characterize the distribution of protein functions in sequence space, we used a comprehensive protein dataset of 39,529 sequences that adopt 457 single-domain structures. In the following, we refer to them simply as structures. The functions we consider are based on the enzyme commission (EC) [42] classification, which distinguishes four different hierarchical levels of enzyme function. The top level comprises six enzyme classes, namely oxidoreductases, transferases, hydrolases, lyases, isomerases and ligases. Each class is subdivided into three further hierarchical levels whose interpretation differs among classes. In this classification system, individual enzymes are assigned a four-digit number where each digit reveals increasing details about enzyme function. For example, the enzyme tryptophan synthase with EC number 4.2.1.20 is a lyase that catalyzes the conversion of indole and serine to tryptophan. Although the EC classification has well-known limitations (eg. see [30]), it is the best-established and most widely used system for classifying enzymes, which are the most prominent protein class. (By March 2010, 57 percent of proteins in the Protein Data Bank [45], a repository of protein structure information, have at least one enzymatic function). For our data set, the bottom, finest-grained level of this classification comprises 1,343 different enzymes. For this data set, Figure S1a shows the distribution of the number of sequences per structure, and Figure S1b shows the number of sequences per function.

Although our data set may seem enormous, we note that it still represents a very sparse sampling of sequence space. For example, approximately 60 percent of functions are represented by fewer than 10 sequences per function. Also, two proteins with the same structure and/or function in our data are typically highly divergent, with a median amino acid divergence of no less than 55 percent (Figure S2a and S2b).

### Most enzymatic functions are associated with few structures

**Figure 1:**
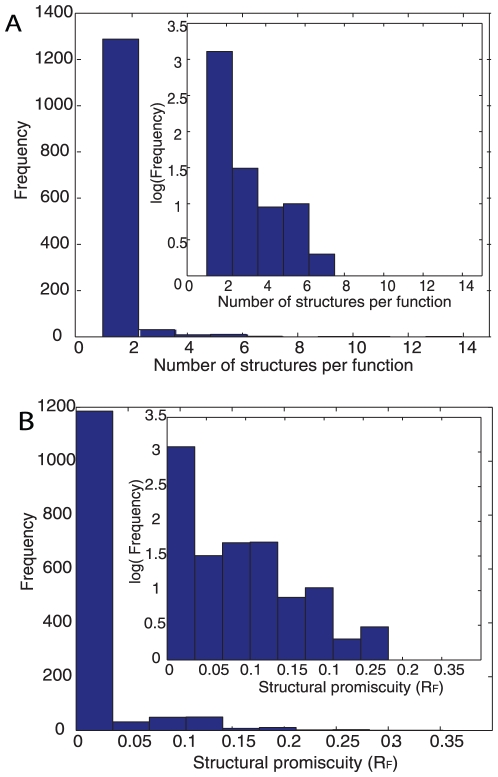
Distribution of structures over functions. (**a**) *Distribution of the number of structures associated with a particular function.* The total number of different structures (457) in our dataset composed of 39.529 sequences are classified according to the enzyme function that they perform and counted (min = 1 ; max = 14 ; mean = 1.2). The inset shows the same distribution, but with a log_10_-transformed vertical axis. (**b**) *Distribution of structural promiscuity*. Structural promiscuity (R_F_) is an entropy-like measure (see main text) calculated from the distribution of enzyme functions over different protein domains. The data shown is based on the finest-grained, fourth level of the EC hierarchy. (min = 0.0; max = 0.35; mean = 0.01).

Any given function in our data set may be carried out by proteins with only one structure, or by multiple different structures. We call the latter kind of function *structurally promiscuous*, because it is not tied to any one structure. Figure 1a shows a histogram of the number of structures associated with a function for the 1,343 lowest level enzymatic functions we discuss here. This distribution is highly skewed, with 86 percent of the functions carried out only by one structure and three maximally promiscuous functions carried out by 9, 11 and 14 structures, respectively. These functions are RNA polymerase (EC = 2.7.7.6); cytochrome oxidase (EC = 1.9.3.1) and DNA polymerase (EC = 2.7.7.7). Figure S3 shows that the distribution remains skewed if we control for the number of sequences known per structure.

We next extended previous work [30] by defining a measure R_F_ of the promiscuity of any given function. We focus on only those sequences that perform a given function *F*. For any given protein structure *i* (out of *N* total structures), we denote as *f(i)* the fraction of sequences among all proteins that perform the function F and fold into structure *i*. The sum of the *f(i)*'s over all structures will add to one. The Shannon entropy of the distribution of the non-zero *f(i)*'s is given by 
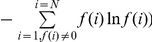
, where ln denotes the natural logarithm. The maximal value of this entropy is ln N, which is attained if every structure is equally likely to perform the function F. Its minimal value of zero is reached if the function is carried out by only one domain k, such that *f(k)* = 1 and all other *f(i)* = 0. These observations motivate the definition of structural promiscuity as 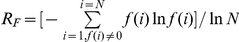, which is an entropy normalized to the interval zero (low promiscuity) and 1 (highest promiscuity). R_F_ adopts its minimum for functions associated only with a single structure. It would attain a maximum for a function that is equally likely to be performed by any structure. (Such a function may not exist.) Figure 1b shows the distribution of R_F_. This distribution is again highly skewed, with a minimum of 0 for 1,161 (86 percent) of functions that are executed only by single domains. The maximal value observed is 0.35. This highest value is attained by DNA-polymerases (EC.2.7.7.7), which are well known to be structurally diverse [46]. It is followed by type II restriction enzymes (rank 2) and ubiquitin carboxyl-terminal hydrolases (rank 3). Table 1 shows the ten most structurally promiscuous enzyme functions. We note that this measure of promiscuity R_F_ weights different structures according to the fraction of known sequences adopting them. It can thus give different results from simpler measures based on counting the number of sequences or structures per function.

**Table 1:** The ten most structurally promiscuous functions.

	EC number	N structures	*R_F_	Catalytic activity
1	EC = 2.7.7.7	14	0.35	DNA-directed DNA polymerase.
2	EC = 3.1.21.4	7	0.29	Type II site-specific deoxyribonuclease
3	EC = 3.1.2.15	6	0.26	Ubiquitin thiolesterase.
4	EC = 1.6.5.3	6	0.26	NADH dehydrogenase (ubiquinone).
5	EC = 2.7.7.48	6	0.25	RNA-directed RNA polymerase.
6	EC = 2.7.7.49	5	0.22	RNA-directed DNA polymerase.
7	EC = 1.14.13.39	4	0.22	4-hydroxyphenylacetate 3-monooxygenase.
8	EC = 3.1.3.2	6	0.21	Acid phosphatase.
9	EC = 2.5.1.18	4	0.20	Glutathione transferase.
10	EC = 2.7.7.6	9	0.20	DNA-directed RNA polymerase.

* (R_F_). Structural promiscuity. (See main text).

The distributions we just presented may reflect underlying properties of sequence space, but also results of biases in existing knowledge about different structures or functions. The most obvious such bias comes from the extent to which different structures and functions have been characterized. It is reflected in the different numbers of sequences that are known for them. Figure S4a and S4b shows that this amount of information can affect estimates of the structural promiscuity of a given function. The figure demonstrates that both the number of structures known to carry out a given function, and the structural promiscuity of a function increase with the number of sequences that are associated with the function. These observations suggest that low structural promiscuity of a function may be more apparent than real, and that promiscuity will increase as more proteins with a given function become characterized.

To summarize our analysis so far, relatively few functions are carried out by multiple structures, but this number would increase as more protein sequences will become characterized. In the supplementary material (File S1), we extend this analysis to the highest level of the EC hierarchy (Figures S5, S6, S7, S8, S9), where we observe similar patterns. In addition, extending previous work [30], we also analyze the distribution of the number of functions per structure (Figures S7). This distribution is similarly skewed, with most structures having single functions, and a minority of structures adopting multiple functions.

### Phenotype neighborhoods

**Figure 2:**
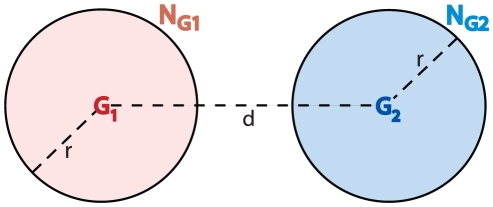
Genotype neighborhoods. Illustration of genotype neighborhoods by a schematic two-dimensional projection of protein sequence space. The neighborhood of a genotype (N_G1_(r) ) is defined as the set of all the genotypes found at a sequence distance equal or shorter than a radius (r) from the genotype of interest. Two such neighborhoods may contain different sets of functions, S_1_ and S_2_, respectively. We define the fraction of functions unique to a neighborhood as F_u_ : = (|S_1_|+|S_2_|−2|S_1_∩ S_2_|)/ |S_1_ ∪ S_2_|.

Thus far, we have examined global aspects of the organization of enzymatic functions, disregarding where the proteins carrying out these functions occur in sequence space. We next turn to a more local analysis that focuses on different neighborhoods of sequence space. We define a neighborhood N_G_(r) of a protein sequence (genotype) *G*, as the set of sequences that differ in no more than a number or percentage *r* of its amino acids from *G* itself. Put differently, a neighborhood N_G_(r) is a ball of radius *r* around *G*. With this notion in hand, we ask whether different neighborhoods differ in the kinds of functions they contain. That is, consider two protein sequences G_1_ and G_2_ with sequence distance *d*, and the neighborhoods N_G1_(r) and N_G2_(r) around them (with some given radius *r*) (Figure 2). The neighborhood of G_1_, N_G1_(r) contains sequences that carry out some set S_1_ of enzymatic functions. Similarly, N_G2_(r) contains sequences that carry out some set S_2_ of enzymatic functions. The number of functions that occur in both neighborhoods equals |S_1_ ∩ S_2_|, where |X| denotes the number of elements in a set X. The set of all functions that are found in at least one of the two neighborhoods is (S_1_ ∪ S_2_). We define the fraction of functions that occur in the neighborhoods of one but not the other sequence as F_u_ : = (|S_1_|+|S_2_|−2|S_1_∩ S_2_|)/ |S_1_ ∪ S_2_|. For brevity, we will refer to it as the fraction of functions unique to a neighborhood. This does not mean that these functions occur nowhere else in sequence space. They just do not occur in the other neighborhood examined. F_u_ depends on the distance *d* between G_1_ and G_2_ and on the neighborhood radius *r*. We explore this dependency below.

### Different genotypic neighborhoods contain highly diverse functions

**Figure 3:**
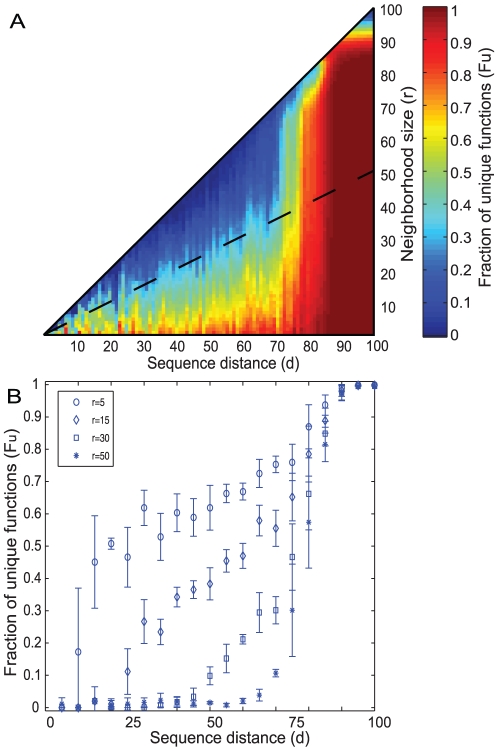
Different genotypic neighborhoods contain highly diverse functions. (**a**) The figure shows a heatmap of the fraction of unique functions (F_u_) at different combinations of neighborhood radii (r) and sequences distances (d). The dataset analyzed here is based on 10 random subsets of 28,862 sequences from our original data, where we required that each sequence in each subset is longer than 100 amino acids. (The sequences in each subset adopted, on average 337 structures and perform 1,036 different enzyme functions.) From each of these 10 subsets, we then chose 10^5^ pairs of sequences at random, and computed their values of *r*, *d*, and F_u_. We repeated this random selection of 10^5^ sequence pairs *n* times, until the results no longer changed. For the dataset of the figure, this convergence occurred around *n* = 10, but data are shown for *n* = 100. The heatmap shows the average values across the 10 samples observed for each combination of distance and radius. (**b**) Fraction of unique functions *F_u_* versus sequence distance (expressed in percent) at constant neighborhood radii, as shown in the legend. Due to the sparsity of data, we grouped values into 20 different distance bins, each spanning d = 5. Error bars represent standard errors calculated for each of these 20 bins.

Figure 3a shows a heat-map of the fraction F_u_ of functions unique to a sequence neighborhood, for our entire data set, and for sequences G_1_ and G_2_ whose distances *d* vary, as well as for sequence neighborhoods of various sizes *r* (smaller than *d*). The region where the two neighborhoods do not overlap, that is, where *r*<*d*/2, is indicated in the figure by a dashed line. For the data in this figure, we chose the neighborhood centers G_1_ and G_2_ regardless of the structure and function of G_1_ and G_2_. Perhaps of the greatest interest are neighborhoods with small radius *r*. They contain functions that can be reached via a small number of changes from its center G_i_.

Two general observations emerge from the figure. First, at any neighborhood size *r*, the fraction of unique functions increases rapidly with the distance between the neighborhood centers G_1_ and G_2_. For a select number of sizes *r*, this relationship is shown also in Figure 3b, which displays F_u_ as a fraction of the sequence distance between G_1_ and G_2_. (The large standard deviations of the data at low values of *d* reflect the very sparse sampling of sequence space at low *d*.) For example, if two different sequences G_1_ and G_2_ of length 100 amino acids differ at only 20 percent of their amino acids, their respective neighborhoods of radius five (which correspond to sequences differing from them in no more than five percent of their amino acids) have merely 50 percent of their functions in common (Figure 3b). In other words, fifty percent of these functions are reachable from one sequence (by no more than five amino acid changes), but not from the other. More generally, small neighborhoods of two distant proteins will generally contain very different functions.

The second general feature occurs at distances between G_1_ and G_2_ that exceed d = 80. Here, the fraction of unique functions F_u_ rapidly increases to a value close to one, regardless of the neighborhood radius. This means that neighborhoods that are very far apart in sequence space contain mostly different functions. We explain below that this feature arises from the fact that highly dissimilar proteins with the same structure, proteins that are not from the same family (d larger than 80 percent) generally have different functions.

### Different genotypic neighborhoods of proteins with a given structure contain highly diverse functions

**Figure 4:**
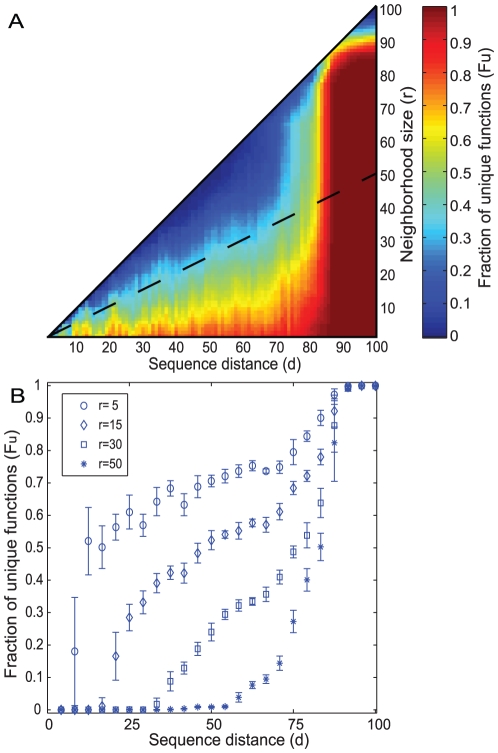
Genotypic neighborhoods of the TIM barrel domain. The figure shows the dependency between the radius and distance of genotype neighborhoods, and the fraction F_u_ of functions unique to one neighborhood, for sequences adopting the TIM barrel domain (see Methods). (**a**) Heatmap of the fraction of unique functions (F_u_) at different combinations of neighborhood radii (*r*) and sequences distances (*d*). We analysed these 4,132 sequences exhaustively. That is, for all possible pairwise sequence comparisons we computed their values of r, d and F_u_. The heatmap shows values of F_u_ at each combination of *d* and *r*. (**b**) Fraction of unique functions versus sequence distance (expressed in percent) at constant neighborhood radii, as shown in the legend. Due to the sparsity of data, we grouped values into 20 different distance bins, each spanning d = 5. Error bars represent standard errors calculated for each of these 20 bins.

The previous analysis focused on the distribution of functions in different sequence space neighborhoods, regardless of the structure or function of the proteins G1 and G2 in the neighborhood centers (Figure 2). We next asked whether similar distributions also exist if G_1_ and G_2_ (Figure 2) have the same structure. This is of course only possible for structures for which many sequences are available. The structure with most associated sequences in our dataset is the TIM barrel. It is represented by 4,132 sequences. These 4,132 sequences carry out 53 different enzymatic functions that cover 5 out of the 6 EC major classes and are widely spread through sequences space (Figure S10). Figure 4a shows, analogous to our analysis above, the fraction of unique enzyme functions (F_u_) found in pairwise comparisons of different neighborhoods in sequence space, when considering only sequences known to fold into the TIM barrel domain. The qualitative features we observed above are also present for the TIM barrel domain. First, the fraction of unique functions increases with increasing sequence distance of the neighborhood centers G_1_ and G_2_ (Figure 4b). Second, at large distances of G_1_ and G_2_, most functions are unique, regardless of the neighborhood radius *r*.

To exclude the possibility that these observations are peculiarities of the TIM barrel domain, we carried out independent analyses for those 36 structures for which the most sequences were available. Together, they comprise a total of 18,117 sequences with lengths ranging from 100 to 400 amino acids, and span 434 enzymatic functions covering all 6 EC classes. In lieu of presenting 36 plots, figure S11 shows data averaged over all 36 structures. Its panels show the fraction F_u_ of unique functions and how it depends on sequence distance *d* and neighborhood radius *r*, exactly as for Figures 3a and 3b. Distances and radii are shown as percentages of total protein length. The figure shows that these 36 structures have properties qualitatively similar to that of the TIM barrel, except that the dramatic increase in F_u_ occurs over a broader range of sequence distances *d* (between ca. 70 and 90 percent, Figure 3a). This observation can be explained if different structures differ in the divergence that two sequences encoding them typically have. Figure S3b shows that this is indeed the case. It is based on the 337 structures that have more than one sequence in our data, and shows that the divergence of these sequences varies broadly around a large median of 92 percent. (For the TIM barrel domain, the maximal distance among sequences is 100%.)

### Neighborhood diversity in functions depends on functionally versatile protein families

Thus far, we saw that the fraction of unique phenotypes increases with increasing distance of two genotypic neighborhoods, regardless of whether these neighborhoods center on proteins with the same structure (Figures 3 and 4) or on proteins with different structure (Figure S11). Our next analysis shows that this high neighborhood diversity comes from the fact that proteins in a given protein family can have multiple functions. Recall that a protein family, as used here, is a set of proteins with the same structure, and a sequence distance lower than 70 percent. Figure S12 shows that the sequences adopting any one structure often fall into multiple families.

If neighborhood diversity depends on functional diversity of proteins in the same family, then an analysis of this diversity, but for a subset of protein families with only one function per family should lead to a fundamentally different result from that observed in Figures 3, 4, and S11. We thus repeated our analysis of functional diversity for the TIM barrel structure, but for a subset of its protein families that carry out only single functions (Figure S13). The analysis shows that different neighborhoods now contain identical functions for all neighborhood centers with less than d = 80 percent divergence, which is the divergence of these TIM barrel families. Functional diversity of different small neighborhoods thus disappears, if we consider mono-functional protein families. At d>80 percent, however, neighborhood divergence becomes close to maximal, as in our earlier analysis. This is because protein pairs at this distance fall into different families, and typically have different functions. For example, a comparison of all pairs of monofunctional protein families within the TIM barrel domain shows that only 1.6 percent of these pairs have the same function. This pattern also holds for our whole data set, where 75 percent (1,162) of the protein families perform single functions and only 0.1 percent of the family pairs (with the same or different structure) have the same function.

In sum, if protein structure equaled function, then all but the most distant genotypic neighborhoods would be functionally homogeneous. Functional neighborhood diversity emerges from the multifunctionality of structures.

## Discussion

In sum, our large data set of more than 30,000 protein sequences with known structures and enzymatic functions gives rise to three general observations. First, as shown previously [30], different functions are carried out by different numbers of sequences and structures. Second, most functions are restricted to single structures, but some can be carried out by many structures. Relatedly, most protein families are associated with only one function, as was also shown previously based on fewer data [30]. Third, and most important, different genotype neighborhoods tend to contain a different spectrum of functions, whose diversity increases with increasing distance of these neighborhoods in sequence space.

One would be more likely to find functions that can be executed by many structures in sequence space than those carried out by only one structure, because, with possible exceptions, such functions would also be carried out by more sequences. While it is tempting to interpret the first and second observation above as firm evidence that different functions differ in the proportion of sequences that can perform them, this evidence has to be taken with a grain of salt. First, some functions may be needed by few organisms or in few environments. Fewer proteins carrying out these functions may exist than for other, more generally important functions. Second, the data we analyze is not a random sample of sequence space. Some enzymes may be better studied than others, for reasons of their medical importance, or merely by historical accident. Fundamentally, every existing sample of proteins is subject to these problems. However, we can get hints about intrinsic differences among functions in the number of associated sequences if we study the number of functions per structure, in particular if we control for the different number of sequences per structure. Our analysis above showed that the number of structures per function has a nonuniform distribution, even after controlling for the number of known sequences for each structure (Figure S3). This observation hints that some functions may indeed be more frequent in sequence space than others.

In support of this notion, in vitro selection experiments on random polymers and mutagenesis experiments indeed suggest that proteins with different functions may occupy different proportions of sequence space [13], [14], [35]. For example, Taylor et al (2001) explored random libraries of a helical bundle chorismate mutase. They found previously unidentified residues involved in the formation of the enzyme active site. The authors estimate a probability of the order of 10^−23^ of finding this functional enzyme using the same fold in sequence space [13]. Axe [14] examined the probability to find an enzyme in sequence space. His results based on non-biased random libraries of beta-lactamase suggest that this catalyst is rare, with an occurrence probability of 10^−64^. He suggests that the overall probability of finding any functional protein in the sequence space is as low as 10^−77^. Yet another study used phage display to examine the probability to find ATP binding proteins from a random sample of sequence space regardless the fold [47]. Its authors estimated a probability of 10^−11^ to find an ATP binding protein, suggesting that a protein with this function could be found easily in a random search of the sequence space. Although estimates like these depend on various factors, including the length of the proteins considered, they suggest that the probability to find a functional protein in sequence space can vary broadly.

Our most important, third observation, the high phenotypic diversity of different neighborhoods in sequence space, has obvious implications for the evolution of novel protein functions. If a protein performs an essential function, then this function needs to be preserved over time. This typically means that the protein's structure will also be preserved, because changes in protein structure typically require changes in many amino acid sequences and would thus not preserve function [48], [49]. Populations of organisms are subject to mutations that change individual amino acids. They may also be subject to recombination between homologous proteins of the closely related individuals within a population. This means that proteins that preserve their function change their genotype gradually over time. In other words, they drift through the function's genotype network, which can extend very far through genotype space [50], [51]. In doing so, they explore different regions of genotype space, all the while preserving their function [52]. Consider now two proteins with the same function but in different parts of this space. If their neighborhoods typically contained the same spectrum of functions, the exploration of a genotype network would not aid in their exploration of novel functions. If conversely, these neighborhoods differ in the function they contain, the exploration of a genotype network may be crucial to explore new functions, some of which may become evolutionary innovations. This is exactly the property we found here. That is, by exploring a genotype network, proteins can explore ever-changing sequence neighborhoods, and an ever-changing spectrum of novel enzymatic functions.

The functional diversity of different neighborhoods we observe is caused by differences in the apparent structural promiscuity of a particular function. That is, if any one function could only be carried out by one structure, then different neighborhoods of two proteins with the same structure or function would not contain diverse novel functions. This observation underscores the importance of studying the organization of protein functions in sequence space independently from the organization of structures.

The phenotypic diversity of different neighborhoods in sequence space also has a flip side: It means that not all protein functions occur in every neighborhood of sequence space. In other words, the evolution of novel protein functions is *constrained* by an individual or a population's location in sequence space. A consequence of such constraints is evolutionary stasis, where genotypes but not phenotypes in a population change while the population explores a genotype network. Such stasis is interrupted by the discovery of novel phenotypes when a population arrives at a neighborhood where such novel phenotypes are found. In other words, evolutionary constraints can lead to patterns of episodic evolution, where periods of stasis are interrupted by discoveries of novel phenotypes. Such episodic evolution has been documented in systems ranging from evolving RNA molecules to macroscopic traits in the fossil record [53]–[57]. Although to our knowledge no demonstration of episodic evolution is known for protein functions, our observations suggest that it will also be widespread for proteins.

The causes of evolutionary constraints on the acquisition of new phenotypes are the subject of a broad literature and wide debate, particularly among students of organismal development and its evolution [58]–[62]. In this literature, the causes of constrained evolution are often unclear, because the relationship between genotype and phenotype is very complex for the macroscopic traits that development creates. This relationship involves many genes, and is thus incompletely understood. Protein functions are simpler, molecular phenotypes, which allow us to circumvent these complexities. For them, constrained evolution emerges from the organization of phenotypes in a genotype space. These observations, if generalizable to more complex traits, imply that we need to understand the organization of such complex traits in their genotype space, before we can hope to understand constrained evolution well.

Our study also reveals similarities and differences between the space of protein structure and functions when mapped onto sequence space (Figure 3, S2 and S13). As previous studies also showed, structures are highly conserved in sequence space [63], [64]. For example, pairs of sequences may diverge by more than 95 percent and still fold into the same structure [11].

Early bioinformatic analyses suggested that the organization of protein functions was similar to that of protein structures [26]–[28], but later work showed that functions and structures have different organization in sequence space and functional annotation can not only rely on sequence similarity [32].

Here we observed that new functions are encountered at varying sequence distances as proteins diverge in sequence space, and that this property can be attributed to the fact that some protein families perform multiple functions. While for short distances in sequences space this diversity is moderate, it increases at larger distances and once the structure conservation threshold (i.e. 70 to 80 percent sequence identity) is crossed, we observed an explosion in the accessibility of new structures [11], [63], and consequently an enormous increase in functional diversity (Figure 3,4 and S13).

The characterization of protein sequence spaces with large but heterogeneous biological data like ours has several caveats. First, different proteins have different lengths, and thus exist in genotype spaces of different dimensions. To compare neighborhoods, however, we need to embed proteins within a genotype space of a given dimension. For our analysis, we solved this problem by restricting some analyses to proteins of similar length, and by focusing others on subsets of multiple sequence alignments that have the same lengths. This amounts to projecting genotype spaces of higher dimensions onto lower-dimensional spaces. It reduces the size of our data set, an unavoidable consequence of this procedure.

A second problem is posed by the vast size of genotype space. Our data set is very large, but even data sets many orders of magnitudes larger than ours would sample such a space only very sparsely. The limited functional diversity of the smallest sequence neighborhoods we examine likely results from this sparsity.

Third, our data set is a non-random sample of sequence space, with many biases whose extent is unknown. Some of the properties we study, such as the structural promiscuity of a function, are not easy to infer from such a data set, nor can they be inferred from models of protein folding such as lattice proteins, because such models are ill-suited to study protein function. We will not be able to characterize these properties rigorously until we are able to generate random samples in sequence space of proteins with a given function, which requires computational tools that are not yet within reach.

We note in closing that the property central to our study - the phenotypic diversity of different neighborhoods - is not likely to be strongly affected by biases in our data. Specifically, we showed that different phenotypic neighborhoods contain different phenotypes, largely because multifunctional protein structures exist. In our data, such multifunctional structures comprise a minority of structures. This observation may well be an artifact of a biased sampling of sequence space. If we had the same, large amount of sequence information for all structures, we might find most structures to be functionally versatile; and we might find most functions to be executable by multiple structures. If anything, the functional diversity of different neighborhoods in sequence space would thus increase. Thus, the very feature that both facilitates evolutionary exploration of novel functions and causes their constrained evolution is probably a generic property of protein sequence space.

## Supporting Information

File S1 We extend earlier work on statistics of protein functions, specifically: 1) the number of structures per function for the six top-level EC functions; and 2) the numbers of sequences per function against the number of structures per function and the promiscuity of a function for the six major enzyme classes EC1 through EC6.
(0.06 MB DOC)

Figure S1 Distribution of the number of sequences per structures and per functions. (a) Distribution of the number of sequences per structure. Histogram of the total number of sequences per structure (min = 1; max = 4.134; mean = 84). (b) Distribution of the number of sequences per function. Histogram of the total number of sequences per function, according to the EC classification finest-grained level (min = 1; max = 578; mean = 29). Distributions are based on our data set composed of 39,529 sequences, 457 structures and 1,343 enzymes types.
(1.05 MB EPS)

Figure S2 Distribution of distances between sequences. (a) Distribution of distances between all sequence pairs with the same structure and function. (min = 0; max = 100; median = 55; mean = 54). The distribution shows values of all against all pairwise distances between sequences that fold into the same structure and are classified under the same enzyme function. (b) Distribution of distances between all sequence pairs with the same function. (min = 0; max = 100; median = 56; mean = 57). The functional annotation is based on the finest-grained level of the EC hierarchy. (c) Distribution of distances between all sequence pairs with the same structure. (min = 0; max = 100; median = 92; mean = 86). The data for these distributions was generated as follows. From our original data composed of 39,529 sequences, 457 structures and 1,343 enzyme functions, we extracted 10 independent samples of random sections from those multiple sequence alignments that comprised at least 100 amino acids. We required each random section to comprise 100 amino acids. These 10 samples were on average composed of 28,862 sequences, 337 structures and 1,036 enzyme functions. We then chose, from each of the 10 random samples, 10^7^ sequence pairs with identical structure and/or function at random, and calculated their pairwise distances. Error bars indicate standard errors of the mean over the 10 independent samples.
(0.99 MB EPS)

Figure S3 Distribution of the number of structures per function, corrected for the number of sequences. For this figure we used the original dataset of 39,529 sequences, 457 structures and 1,343 enzyme functions. We determined, for each structure i, the fraction f_i_ of sequences adopting this structure. For each function, we then determined all structures that are associated with this function, and averaged the corresponding values of f_i_. The panel shows a histogram of these averages, for all 1,343 enzymatic functions.
(0.01 MB EPS)

Figure S4 Structures per function versus sequences per function. Associations between number of sequences and structures per protein function at the fourth, finest-grained (a,b) and the first, coarsest level (c,d) of the EC hierarchy. For the first analysis (panel a and b), we classified the 39,529 sequences of our original data set according to their enzyme functions and compared the number of sequences per function with the number of structures per function. There are a total of 457 structure and 1,343 functions at this level. For the second analysis of the top-level EC functions, the 39,529 sequences fall into only 6 different enzyme types. While it is difficult to make statistically rigorous statements based on so few functions, we nonetheless wanted to understand how sensitive our observations in panel c) and d) were to the structure of our data. To this end, we extracted random samples of 10^4^ sequences from our data set and classified them according to the 6 top EC-levels. We repeated this procedure 10^5^ times and compare the statistics of the averaged values obtained from the sampling with the statistics observed for the whole data set (without sampling). Plots show the means over the sampling and error bars the standard deviations. (a) Scatterplot of the number of sequences per function against the number of structures per function. Spearman rank's correlation r = 0.29 (P<E-50). (b) Scatterplot of the number of sequences per function versus structural promiscuity. Spearman rank's correlation r = 0.27 (P<E-50). (c) Scatterplot of the number of sequences per function against the number of structures per function at the top level of the EC hierarchy. Spearman rank's correlation r = 0.92 (P<0.01). Spearman rank's correlation of the complete data set (without sampling) is r = 0.94 (P<0.01). (d) Scatterplots of the number of sequences per function at the coarsest level of the EC hierarchy versus structural promiscuity. Spearman rank's correlation r = 0.92 (P<0.01). Note the decadic logarithms on the vertical axes of all plots. Spearman rank's correlation of the complete data set (without sampling) is r = 0.77 (P<0.1).
(1.70 MB DOC)

Figure S5 Distribution of structures over functions at the top level of the EC hierarchy. (a) Number of structures per enzyme class at the first (top) level of the EC hierarchy. For this figure, we grouped the total number of different structures (457) in our dataset composed of 39,529 sequences are classified according to the enzyme function that they perform (min = 28; max = 188; mean = 100). (b) Structural promiscuity at the first level of the EC hierarchy. Structural promiscuity (R_F_) is an entropy-like measure (see main text of the Supplementary Material) calculated from the distribution of the EC top-level types of enzyme functions over different protein structures (min = 0.32; max = 0.57; mean = 0.49).
(0.88 MB EPS)

Figure S6 Distribution of functions over structures.(a) Distribution of the number of functions per structure at the fourth (finest grained) level of the EC hierarchy. (min = 1, max = 103). (b) Distribution of functional versatility (V_S_) at the fourth level of the EC hierarchy. Functional versatility (V_S_) is an entropy-like measure (see main text) calculated from the distribution of structure domains over different enzyme functions at the bottom level of the EC hierarchy. (min = 0, max = 0.53). For the data in these panels, we classified the total number of different enzyme functions (1,343) according to the structures that carry them out (457).
(1.04 MB EPS)

Figure S7 Distribution of functions over structures at the coarsest level of the EC hierarchy.(a) Distribution of the number of functions per structure at the coarsest level of the EC hierarchy. The data is based on the total number of 6 different enzyme types at the first, coarsest level of the EC hierarchy in our dataset of 39,529 sequences and 457 strcutures. For the plot, we classified each sequence according to its structure and function. (min = 1, max = 5;). (b) Distribution of functional versatility (V_S_) at the coarsest level of the EC hierarchy. Functional versatility (V_S_) is an entropy-like measure (see text) calculated here from the distribution of structure domains over different enzyme functions at the first, coarsest level of the EC hierarchy (min = 0, max = 0.76). The inset show the same data, but with a log_10_-transformed vertical axis.
(0.86 MB EPS)

Figure S8 Sequences per structure versus the distribution of functions. (a) Scatterplot of the number of sequences per structure against the number of functions per structure. The association between number of sequences and enzyme functions per structure domain is shown for the fourth (finest grained) level of the EC hierarchy. Spearman rank's correlation r = 0.57 (P<E-50). (b) Scatterplot of the number of sequences per structure versus functional versatility. The same dataset described in panel (a) is used to examine the association between number of sequences (39,529) and the functional versatility (V_S_) per structure domain. Spearman rank's correlation r = 0.51 (P<E-50). For the data in this figure, we classified the number of sequences (39,529) and enzyme functions (1,343) according to their structure (457). Note the log10-transformed horizontal axes.
(1.33 MB EPS)

Figure S9 Scatterplot of the number of sequences per structure. Associations between numbers of sequences and functions per structure are shown at the first, coarsest level of the EC hierarchy. We classified the 39,529 sequences according to their 457 structures and compared the number of sequences per structure with (a) the number of functions per structure and (b) functional versatility (V_S_). For the first analysis (panel a), we classified the number of functions (at the coarsest level of the EC hierarchy) per structure in our dataset and the corresponding number of sequences folding into those structures (Spearman rank's correlation r = 0.43; P<E-50), Error bars represent the standard error over the number of sequences per structure. The second panel (b) shows a scatterplot comparing the number of sequences per structure (log_10_-transformed) and V_S_ per structure (Spearman rank's correlation r = 0.42; P<E-50).
(0.98 MB EPS)

Figure S10 Principal Component Analysis (PCA) of the TIM barrel main homologous superfamily (the aldolase I superfamily). For this analysis, we first constructed a multiple sequence alignment of the aldolase I superfamily (CATH code: 3.20.20.70), using the program clustalw, and allowing no more than 10 percent gaps in the alignment. The resulting multiple sequence alignment is composed of 4,132 sequences of length 188 amino acids, and comprises 53 different enzyme functions at the finest-grained level of the EC hierarchy. For subsequent PCA [4], we encoded the sequences in the alignment as numeric strings (21 possible values per amino acid position, including gaps). The panels show the first two principal components (a) and the first and third components (b). The 53 different enzyme functions are color-coded according to the color bar to the right. Note the clear separation of some functions.
(3.82 MB EPS)

Figure S11 Genotypic neighborhoods of proteins with a given structure. The figure shows the dependency between the radius and distance of sequence neighborhoods, and the fraction F_u_ of functions unique to one neighborhood, for sequences folding into 36 different structures. The total set of multiple alignments we used in this analysis comprises a total of 18,117 sequences with lengths ranging from 100 to 400 amino acids, and spans 434 enzymatic functions covering all 6 EC classes. We analysed these sequences exhaustively. That is, for all possible pairwise sequence comparisons we computed their values of r, d and F_u_. The heatmap shows F_u_ values at each combination of d and r, for the 26 structures (a) Heatmap of the fraction of unique functions (F_u_) at different combinations of neighborhood radii (r) and sequences distances (d). (b) Fraction of unique functional F_u_ of unique functions versus sequence distance (expressed in percent) at a given neighborhood radius, as shown in the legend. Due to the sparsity of data, we grouped values into 20 different distance bins, each spanning d = 5. Error bars represent standard errors calculated for these 20 bins. The CATH identifiers of the 36 superfamilies we used in this analysis are listed here: 3.30.70.141; 3.30.420.10; 3.40.50.960; 2.70.40.10; 3.90.45.10; 3.40.50.2020; 3.20.19.10; 3.40.50.1470; 3.40.50.1360; 2.40.10.10; 3.90.1550.10; 3.90.226.10; 3.90.180.10; 3.40.50.880; 3.60.20.10; 3.40.50.620; 3.40.1210.10; 3.40.1160.10; 3.40.50.1240; 3.40.640.10; 3.60.15.10; 3.20.20.60; 3.20.20.70; 3.30.572.10; 3.90.550.10; 1.20.200.10; 3.40.1190.20; 3.30.930.10; 1.10.1040.10; 3.20.20.140; 3.40.50.1820; 3.20.20.210; 3.20.20.150; 3.40.718.10; 3.20.20.80; 1.10.630.10.
(2.31 MB EPS)

Figure S12 Distribution of the number of protein families per structures. (a) Distribution of the number of protein families per structure domain in the whole CATH database. This data is composed of 114,215 protein families grouped into 2,178 structures. (b) Distribution of the number of protein families per structure in our dataset composed of 39,529 sequences and 457 structures. More precisely, the notion of a protein family here corresponds to that of a CATH homologous superfamily (Greene et al, 2007). The insets show the same data, but with a log_10_-transformed vertical axis.
(0.92 MB EPS)

Figure S13 Neighborhood diversity in functions depends on functionally versatile protein families and structures. The figure shows the dependency between the radius and distance of two genotype neighborhoods, and the fraction F_u_ of functions unique to one neighborhood. (a) Heatmap of the fraction of unique functions (F_u_) at different combinations of neighborhood radii (r) and sequences distances (d). The data is based on the major superfamily of the TIM barrel domain, aldolase I (CATH code: 3.20.20.70), which is composed of 4,132 sequences that carry out 53 different enzyme functions (see methods). These sequences can be grouped into 62 protein families. From this data set we selected the 30 protein families that carry out single enzyme functions. These families comprise 2,444 protein sequences and 27 enzyme functions. For all possible sequence pairs in this data set we computed values of d and F_u_ for different values of r. The heatmap shows Fu values over all distance-radius combinations. (b) Fraction of unique functional variations versus sequence distance (expressed in percent) at constant neighborhood radii, as shown in the legend. Due to the sparsity of the data, we grouped values into 20 different distance bins, each spanning d = 5. Error bars represent standard errors calculated for these 20 bins.
(2.09 MB EPS)
