# Supplementary material for: Evolutionary Innovations and the Organization of Protein Functions in Genotype Space
Source: PLoS One. 2010 Nov 30;5(11):e14172. doi: 10.1371/journal.pone.0014172 (PMC2994758; doi:10.1371/journal.pone.0014172)
Supplement: File S1 — We extend earlier work on statistics of protein functions, specifically: 1) the number of structures per function for the six top-level EC functions; and 2) the numbers of sequences per function against the number of structures per function and the promiscuity of a function for the six major enzyme classes EC1 through EC6. (0.06 MB DOC) [file pone.0014172.s001.doc]

**Supplementary Online Material**

*Supplementary Results*

We here analyze the number of structures per function for the six top-level EC functions given in Figure S5a. It can be seen that certain enzyme functions are more common than others. This bias may stem from biases in experimental studies, such as differences in the ease with which soluble proteins crystallize. However, some authors have argued that certain enzyme chemistries would prevail over others due to their requirements in nature (Nagano et al. 2001). We also characterized the structural promiscuity (R_F_) for the six major enzyme classes (Figure S5b). Its distribution is nearly even among classes, with a small excess for hydrolases (EC.3). These are enzymes that cleave molecular bonds using water, and include proteases and lipases. Hydrolases are the most common enzyme class in the EC commission nomenclature, and also have more subclasses than other classes. We note that the frequency of a function on one level does not imply high promiscuity on another level. For example, the most abundant DNA polymerase function we discussed earlier is not a member of the most abundant enzyme class of hydrolases (EC.3). Instead, it is a transferase.

Figure S4c and d plot the numbers of sequences per function against the number of structures per function (panel c), and the structural promiscuity of a function (panel d) for the six major enzyme classes EC1 through EC6. For only six classes, it is difficult to calculate statistically meaningful associations between the numbers of sequences per function, and measures of the structural promiscuity of a given function.

*Most structures carry out few enzymatic functions.*

Our earlier analyses (Figures S1-S5) focused on individual functions, and asked by how many structures they are carried out. In a complementary analysis, we now focus on individual structures, and ask about the number of functions they carry out. This analysis extends earlier work on statistics of protein functions (Martin et al. 1998; Nagano et al. 2001; Todd et al. 2001). The result is shown as a histogram for our 1,343 lowest-level enzyme functions in Figure S6a. It shows that most structures (54 percent; 248 structures) carry out only one function. The structure with the largest number of 103 associated functions is the NAD(P) binding Rossmann-like domain.

A complementary measure of the extent to which the same structure carries out different functions considers only sequences that have a given structure. It denotes by *f(i)* the fraction of these sequences with function *i*, and defines a normalized measure of how functionally versatile a structure is. We call this measure the *functional versatility* (*V_S_*) of a structure, and define it, exactly as our above measure of the structural promiscuity of a function, as $V_{S}=[-\sum_{i=1,f(i)\neq0}^{i=N} f(i)\ln f(i)]/\ln N$. This measure is again akin to a normalized entropy. It ranges from a value of zero if all sequences with this structure carry out a single function, to a maximal value of one if *f(i)* has the same value for all *i* functions, that is, if a randomly chosen sequence with this structure were equally likely to adopt any function. Figure S6b shows the distribution of this measure for our 457 structures. This distribution is again is highly skewed. It has its lowest value of 0 for 248 structures associated with only a single function, as well as a maximum value of 0.53, which occurs for the TIM barrel domain.

Figure S7 shows analogous analyses, but only for the 6 coarsest levels of the EC hierarchy. A major proportion of the structures (79 percent) carry out functions that fall into a single functional class. Only two of the 457 structures are associated with functions in 5 of the 6 major enzyme classes. These are the Rossmann and the TIM barrel homologous superfamilies. Figure S7b shows the distribution of functional versatility V_S_ for the six top-level enzyme functions. As expected, this distribution is highly skewed.

As in our other analyses, we wanted to explore the extent to which these distributions result simply from biases in available amounts of sequence information. Figure S8 shows, for 1,343 enzymatic functions, scatterplots of the number of sequences per structure against the number of functions per structure (panel a), and against the functional versatility of a structure (panel b). The figure shows that both the number of functions per structure, and the apparent versatility of a structure increase with the number of sequences that are associated with a function. These observations again suggest that apparent low functional versatility of a structure may result from a limited number of characterized sequences with this structure. Figure S9a and b show analogous scatterplots for the six top-level enzymatic functions. A positive association is also evident here.

***References***

Jolliffe IT. 2002. Principal component analysis. Springer Verlag, New York, Inc.

Martin ACR, Orengo CA, Hutchinson EG, Jones S, Karmirantzou M, Laskowski RA. 1998. Protein folds and functions. Structure 6:875-884.

Nagano N, Porter CT, Thornton JM. 2001. The (beta-alpha)8 glycosidases: sequence and structure analyses suggest distant evolutionary relationships. Protein Eng. 14:845-855.

Todd AE, Orengo CA, Thornton JM. 2001. Evolution of function in protein superfamilies, from a structural perspective. J. Mol. Biol. 307:1113-1143.

**Supplementary Figures**

**Figure S1**. *Distribution of the number of sequences per structures and per functions.* (**a**) *Distribution of the number of sequences per structure*. Histogram of the total number of sequences per structure (min=1; max=4,134; mean=84). (**b**) *Distribution of the number of sequences per function*. Histogram of the total number of sequences per function, according to the EC classification finest-grained level (min=1; max=578; mean=29). Distributions are based on our data set composed of 39,529 sequences, 457 structures and 1,343 enzymes types.

**Figure S2**. *Distribution of distances between sequences*. (**a**) Distribution of distances between all sequence pairs with the same structure and function. (min=0; max=100; median=55; mean=54). The distribution shows values of all against all pairwise distances between sequences that fold into the same structure and are classified under the same enzyme function. (**b**) Distribution of distances between all sequence pairs with the same function. (min=0; max=100; median=56; mean=57). The functional annotation is based on the finest-grained level of the EC hierarchy. (**c**) Distribution of distances between all sequence pairs with the same structure. (min=0; max=100; median=92; mean=86). The data for these distributions was generated as follows. From our original data composed of 39,529 sequences, 457 structures and 1,343 enzyme functions, we extracted 10 independent samples of random sections from those multiple sequence alignments that comprised at least 100 amino acids. We required each random section to comprise 100 amino acids. These 10 samples were on average composed of 28,862 sequences, 337 structures and 1,036 enzyme functions. We then chose, from each of the 10 random samples, 10^7^ sequence pairs with identical structure and/or function at random, and calculated their pairwise distances. Error bars indicate standard errors of the mean over the 10 independent samples.

**Figure S3**. *Distribution of the number of structures per function, corrected for the number of sequences.* Here we used the original dataset of 39,529 sequences, 457 structures and 1,343 enzyme functions. We determined, for each structure *i*, the fraction *f_i_* of sequences adopting this structure. For each function, we then determined all structures that are associated with this function, and averaged the corresponding values of *f_i_*. The panel shows a histogram of these averages, for all 1,343 enzymatic functions.

**Figure S4.** *Structures per function versus sequences per function.* Associations between number of sequences and structures per protein function at the fourth, finest-grained (*a,b*) and the first, coarsest level (*c,d*) of the EC hierarchy. For the first analysis (panel a and b), we classified the 39,529 sequences of our original data set according to their enzyme functions and compared the number of sequences per function with the number of structures per function. There are a total of 457 structure and 1,343 functions at this level. For the second analysis of the top-level EC functions, the 39,529 sequences fall into only 6 different enzyme types. While it is difficult to make statistically rigorous statements based on so few functions, we nonetheless wanted to understand how sensitive our observations in panel c) and d) were to the structure of our data. To this end, we extracted random samples of 10,000 sequences from our data set and classified them according to the 6 top EC- levels. We repeated this procedure 10^5^ times and compare the statistics of the averaged values obtained from the sampling with the statistics observed for the whole data set (without sampling). Plots show the means over the sampling and error bars the standard deviations. (**a**) *Scatterplot of the number of sequences per function against the number of structures per function*. Spearman rank’s correlation r=0.29 (P<E-50). (**b**) *Scatterplot of the number of sequences per function versus structural promiscuity.* Spearman rank’s correlation r=0.27 (P<E-50). (**c**) *Scatterplot of the number of sequences per function against the number of structures per function at the top level of the EC hierarchy*. Spearman rank’s correlation r=0.92 (P<0.01). Spearman rank’s correlation of the complete data set (without sampling) is r=0.94 (P<0.01). (**d**) *Scatterplots of the number of sequences per function at the coarsest level of the EC hierarchy versus structural promiscuity.* Spearman rank’s correlation r=0.92 (P<0.01). Note the decadic logarithms on the vertical axes of all plots. Spearman rank’s correlation of the complete data set (without sampling) is r=0.77 (P<0.1).

**Figure S5**. *Distribution of structures over functions at the top level of the EC hierarchy.*  (**a**) *Number of structures per enzyme class at the first (top) level of the EC hierarchy*. For this figure, we grouped the total number of different structures (457) in our dataset composed of 39,529 sequences are classified according to the enzyme function that they perform (min=28; max=188; mean= 100). (**b**) *Structural promiscuity at the first level of the EC hierarchy*. Structural promiscuity (R_F_) is an entropy-like measure (see main text of the Supplementary Material) calculated from the distribution of the EC top-level types of enzyme functions over different protein structures (min=0.32; max=0.57; mean=0.49).

**Figure S6**. *Distribution of functions over structures.* (**a**) *Distribution of the number of functions per structure* *at the fourth (finest grained) level of the EC hierarchy.* (min=1, max=103). (**b**) *Distribution of functional versatility (V_S_) at the fourth level of the EC hierarchy.* Functional versatility (V_S_) is an entropy-like measure (see main text) calculated from the distribution of structure domains over different enzyme functions at the bottom level of the EC hierarchy. (min=0, max=0.53). For the data in these panels, we classified the total number of different enzyme functions (1,343) according to the structures that carry them out (457).

**Figure S7**. *Distribution of functions over structures at the coarsest level of the EC hierarchy.* (**a**) *Distribution of the number of functions per structure at the coarsest level of the EC hierarchy.* The data is based on the total number of 6 different enzyme types at the first, coarsest level of the EC hierarchy in our dataset of 39,529 sequences and 457 structures. For the plot, we classified each sequence according to its structure and function. (min=1, max=5). (**b**) *Distribution of functional versatility (V_S_) at the coarsest level of the EC hierarchy.* Functional versatility (V_S_) is an entropy-like measure (see text) calculated here from the distribution of structure domains over different enzyme functions at the first, coarsest level of the EC hierarchy. (min=0, max=0.76). The inset show the same data, but with a log_10_-transformed vertical axis.

**Figure S8**. *Sequences per structure versus the distribution of functions.* (**a**) *Scatterplot of the number of sequences per structure against the number of functions per structure*. The association between number of sequences and enzyme functions per structure domain is shown for the fourth (finest grained) level of the EC hierarchy. Spearman rank’s correlation r=0.57 (P<E-50). (**b**) *Scatterplot of the number of sequences per structure versus functional versatility.* The same dataset described in panel (a) is used to examine the association between number of sequences (39,529) and the functional versatility (V_S_) per structure domain. Spearman rank’s correlation r=0.51 (P<E-50). For the data in this figure, we classified the number of sequences (39,529) and enzyme functions (1,343) according to their structure (457). Note the log_10_-transformed horizontal axes.

**Figure S9**. *Scatterplot of the number of sequences per structure.* Associations between numbers of sequences and functions per structure are shown at the first, coarsest level of the EC hierarchy. We classified the 39,529 sequences according to their 457 structures and compared the number of sequences per structure with (a) the number of functions per structure and (b) functional versatility (V_S_). For the first analysis (panel a), we classified the number of functions (at the coarsest level of the EC hierarchy) per structure in our dataset and the corresponding number of sequences folding into those structures (Spearman rank’s correlation r=0.43; P<E-50). Error bars represent the standard error over the number of sequences per structure. The second panel (b) shows a scatterplot comparing the number of sequences per structure (log_10_-transformed) and V_S_ per structure (Spearman rank’s correlation r= 0.42; P<E-50).

**Figure S10**. *Principal Component Analysis (PCA) of the TIM barrel main homologous superfamily (the aldolase I superfamily).* For this analysis, we first constructed a multiple sequence alignment of the aldolase I superfamily (CATH code: 3.20.20.70), using the program clustalw, and allowing no more than 10 percent gaps in the alignment. The resulting multiple sequence alignment is composed of 4,132 sequences of length 188 amino acids, and comprises 53 different enzyme functions at the finest-grained level of the EC hierarchy. For subsequent PCA (Jolliffe 2002), we encoded the sequences in the alignment as numeric strings (21 possible values per amino acid position, including gaps). The panels show the first two principal components (a) and the first and third components (b). The 53 different enzyme functions are color-coded according to the color bar to the right. Note the clear separation of some functions.

**Figure S11**. *Genotypic neighborhoods of proteins with a given structure.* The figure shows the dependency between the radius and distance of sequence neighborhoods, and the fraction F_u_ of functions unique to one neighborhood, for sequences folding into 36 different structures. The total set of multiple alignments we used in this analysis comprises a total of 18,117 sequences with lengths ranging from 100 to 400 amino acids, and spans 434 enzymatic functions covering all 6 EC classes. We analysed these sequences exhaustively. That is, for all possible pairwise sequence comparisons we computed their values of r, d and F_u_. The heatmap shows F_u_ values at each combination of *d* and *r*, for the 26 structures (**a**) Heatmap of the fraction of unique functions (Fu) at different combinations of neighborhood radii (r) and sequences distances (d). (**b**) Fraction of unique functional F_u_ of unique functions versus sequence distance (expressed in percent) at a given neighborhood radius, as shown in the legend. Due to the sparsity of data, we grouped values into 20 different distance bins, each spanning d=5. Error bars represent standard errors calculated for these 20 bins. The CATH identifiers of the 36 superfamilies we used in this analysis are listed here: 3.30.70.141; 3.30.420.10; 3.40.50.960; 2.70.40.10; 3.90.45.10; 3.40.50.2020; 3.20.19.10; 3.40.50.1470; 3.40.50.1360; 2.40.10.10; 3.90.1550.10; 3.90.226.10; 3.90.180.10; 3.40.50.880; 3.60.20.10; 3.40.50.620; 3.40.1210.10; 3.40.1160.10; 3.40.50.1240; 3.40.640.10; 3.60.15.10; 3.20.20.60; 3.20.20.70; 3.30.572.10; 3.90.550.10; 1.20.200.10; 3.40.1190.20; 3.30.930.10; 1.10.1040.10; 3.20.20.140; 3.40.50.1820; 3.20.20.210; 3.20.20.150; 3.40.718.10; 3.20.20.80; 1.10.630.10.

**Figure S12**. *Distribution of the number of protein families per structures.* (a) Distribution of the number of protein families per structure domain in the whole CATH database. This data is composed of 114,215 protein families grouped into 2,178 structures. (b) Distribution of the number of protein families per structure in our dataset composed of 39,529 sequences and 457 structures. More precisely, the notion of a protein family here corresponds to that of a CATH homologous superfamily (Greene et al, 2007). The insets show the same data, but with a log_10_-transformed vertical axis.

**Figure S13**. *Neighborhood diversity in functions depends on functionally versatile protein families and structures.* The figure shows the dependency between the radius and distance of two genotype neighborhoods, and the fraction F_u_ of functions unique to one neighborhood. (**a**) Heatmap of the fraction of unique functions (F_u_) at different combinations of neighborhood radii (r) and sequences distances (d). The data is based on the major superfamily of the TIM barrel domain, aldolase I (CATH code: 3.20.20.70), which is composed of 4,132 sequences that carry out 53 different enzyme functions (see methods). These sequences can be grouped into 62 protein families. From this data set we selected the 30 protein families that carry out single enzyme functions. These families comprise 2,444 protein sequences and 27 enzyme functions. For all possible sequence pairs in this data set we computed values of d and F_u_ for different values of r. The heatmap shows F_u_ values over all distance-radius combinations. (**b**) Fraction of unique functional variations versus sequence distance (expressed in percent) at constant neighborhood radii, as shown in the legend. Due to the sparsity of the data, we grouped values into 20 different distance bins, each spanning d=5. Error bars represent standard errors calculated for these 20 bins.
